# Association of Neuroretinal Thinning and Microvascular Changes with Hypertension in an Older Population in Southern Italy

**DOI:** 10.3390/jcm11041098

**Published:** 2022-02-19

**Authors:** Alfredo Niro, Giancarlo Sborgia, Luisa Lampignano, Gianluigi Giuliani, Fabio Castellana, Roberta Zupo, Ilaria Bortone, Pasquale Puzo, Angelo Pascale, Valentina Pastore, Rosa Buonamassa, Roberta Galati, Marco Bordinone, Flavio Cassano, Chiara Griseta, Sarah Tirelli, Madia Lozupone, Vitoantonio Bevilacqua, Francesco Panza, Rodolfo Sardone, Giovanni Alessio, Francesco Boscia

**Affiliations:** 1 Eye Clinic, Hospital “SS. Annunziata”, ASL Taranto, 74100 Taranto, Italy; alfred.nir@tiscali.it; 2 Department of Basic Medical Sciences, Neuroscience and Sense Organs, University of Bari “Aldo Moro”, 70124 Bari, Italy; giuliani_gianluigi@hotmail.it (G.G.); puzopasquale@gmail.com (P.P.); pascaleangelo@gmail.com (A.P.); valentinapastore@hotmail.it (V.P.); rosabuona@hotmail.it (R.B.); robertaga@hotmail.it (R.G.); marcoab313@hotmail.it (M.B.); f.cassano@hotmail.it (F.C.); madia.lozupone@gmail.com (M.L.); f_panza@hotmail.com (F.P.); giovanni.alessio@uniba.it (G.A.); francesco.boscia@uniba.it (F.B.); 3 Unit of Research Methodology and Data Sciences for Population Health, “Salus in Apulia Study”, National Institute of Gastroenterology “Saverio de Bellis”, Research Hospital, 70013 Castellana Grotte, Italy; luisa.lampignano@irccsdebellis.it (L.L.); fabio.castellana@irccsdebellis.it (F.C.); roberta.zupo@irccsdebellis.it (R.Z.); ilaria.bortone@gmail.com (I.B.); chiaragriseta@gmail.com (C.G.); sarahtirelli93@gmail.com (S.T.); rodolfo.sardone@irccsdebellis.it (R.S.); 4 Department of Electrical and Information Engineering, Polytechnic University of Bari, 70126 Bari, Italy; vitoantonio.bevilacqua@poliba.it

**Keywords:** hypertension, older adults, optical coherence tomography, optical coherence tomography angiography, ganglion cell complex, optic nerve head, radial peripapillary capillary

## Abstract

Background: Retinal microvasculature assessment at capillary level may potentially aid the evaluation of early microvascular changes due to hypertension. We aimed to investigate associations between the measures obtained using optical coherence tomography (OCT) and OCT-angiography (OCT-A) and hypertension, in a southern Italian older population. Methods: We performed a cross-sectional analysis from a population-based study on 731 participants aged 65 years+ subdivided into two groups according to the presence or absence of blood hypertension without hypertensive retinopathy. The average thickness of the ganglion cell complex (GCC) and the retinal nerve fiber layer (RNFL) were measured. The foveal avascular zone area, vascular density (VD) at the macular site and of the optic nerve head (ONH) and radial peripapillary capillary (RPC) plexi were evaluated. Logistic regression was applied to assess the association of ocular measurements with hypertension. Results: GCC thickness was inversely associated with hypertension (odds ratio (OR): 0.98, 95% confidence interval (CI): 0.97–1). A rarefaction of VD of the ONH plexus at the inferior temporal sector (OR: 0.95, 95% CI: 0.91–0.99) and, conversely, a higher VD of the ONH and RPC plexi inside optic disc (OR: 1.07, 95% CI: 1.04–1.10; OR: 1.04, 95% CI: 1.02–1.06, respectively) were significantly associated with hypertension. Conclusion: A neuroretinal thinning involving GCC and a change in capillary density at the peripapillary network were related to the hypertension in older patients without hypertensive retinopathy. Assessing peripapillary retinal microvasculature using OCT-A may be a useful non-invasive approach to detect early microvascular changes due to hypertension.

## 1. Introduction

Hypertension, a major risk factor for cardiovascular deaths globally, is expected to affect 1.56 billion adults worldwide by 2025 [1]. It can cause end-organ damage, in the form of cardiovascular disease and nephropathy, resulting in 9.4 million deaths per year around the world [2]. Moreover, many studies have indicated a link between obesity and hypertension, particularly in older people [3]. The prevalence of hypertension rises with age, but it is an easily treatable risk factor for the most prevalent causes of multimorbidity and death in older adults. In particular, hypertension also causes microvascular damage in both the cerebral and retinal circulations [4]. Because the retinal and cerebral vessels share embryological and anatomical characteristics, they may show similar patterns of damage attributable to hypertension.

The value of retinal imaging as a tool to evaluate the ocular effect of hypertension and its importance in making an early prediction of patients’ risk of developing cerebrovascular disease has previously been described [5]. In recent years, hypertensive subjects without hypertensive retinopathy have been shown through optical coherence tomography (OCT) to have a lower thickness of ganglion cell complex (GCC) [6,7] and retinal nerve fiber layer (RNFL) [6,7,8]. Moreover, OCT-angiography (OCT-A) revealed a reduced macular capillary density in hypertensive subjects without related retinopathy [8,9,10]. Previous OCT-A studies have also reported a decreased macular perfusion, along with GCC thinning, in subjects with essential hypertension [9,11]. Those studies were only focused on macular vascular plexi. However, the optic nerve head (ONH) and radial peripapillary capillary (RPC) plexi at the peripapillary site have a key role in the vascular supply of inner neuroretinal layers, including the ganglion cell layer (GCL) and RNFL [12,13]. In recent OCT-A studies, RPC plexus impairment was more evident in older subjects [14], diabetic patients with and without diabetic retinopathy [15], and hypertensive patients with and without retinopathy [8,10].

The aim of the present study was to investigate linear relationships between the retinal features obtained on both OCT and OCT-A scans and blood hypertension, in an older population (aged 65+ years), without related retinopathy, of a cross-sectional study in southern Italy.

## 2. Materials and Methods

### 2.1. Study Population and Design

Data used in the present study were drawn from the population based GreatAGE Study conducted on subjects aged over 65 years, residents of Castellana Grotte, Bari (Puglia Region, southern Italy) participating in the Salus in Apulia Study. The general sampling population was the 19,675 residents listed in the health registry office on 31 December 2014, of whom 4021 subjects were aged 65 years or older. The study design and data collection methods have been described in detail elsewhere [16].

This study presents data from a subpopulation of the study that underwent ophtalmological assessment including OCT and OCT-A (*n* = 731) and were without diabetes mellitus, demyelinating disorders, cardiac diseases. All participants signed informed consent, and the study was approved in 2014 and again in 2019 by the IRB of the National Institute of Gastroenterology “S. De Bellis”, where all the examinations described in this study were performed. The present study adhered to the “Standards for Reporting Diagnostic Accuracy Studies” (STARD) guidelines (http://www.stard-statement.org/, accessed on 1 December 2021, the “Strengthening the Reporting of Observational Studies in Epidemiology” (STROBE) guidelines (https://www.strobe-statement.org/, accessed on 1 December 2021) and is in accordance with the Helsinki Declaration of 1975.

### 2.2. Clinical and Anthropometric Assessment

Height was measured to the nearest 0.5 cm using a wall-mounted stadiometer (Seca 711; Seca, Hamburg, Germany). Body weight was determined to the nearest 0.1 kg using a calibrated balance beam scale (Seca 711; Seca, Hamburg, Germany). BMI was calculated by dividing body weight (kg) by the square of height (m^2^). Waist circumference (WC) was measured at the narrowest part of the abdomen, or area between the tenth rib and the iliac crest (minimum circumference). Office blood pressure measurement was performed four times at 3-h intervals, from 8 am to 5 pm on a single day, following the Hypertension Clinical Practice Guideline [17]. The mean values of the systolic (SBP) and diastolic (DBP) blood pressure for each patient were used in this study. Hypertension was defined as present in participants with elevated BP at the time of examination (SPB ≥ 130 mm Hg or DBP ≥ 80 mm Hg) according to American Heart Association criteria [17]. Education was defined by years of schooling. Smoking was assessed with the single categorized question “Are you a current smoker?”. All participants underwent standardized neuropsychological tests as detailed elsewhere [18] and the Mini-Mental State Examination (MMSE) to assess global cognition [18]. The diagnosis of mild cognitive impairment (MCI) was made according to the Diagnostic and Statistical Manual of Mental Disorders, Fifth Edition (DSM-5) criteria, as detailed elsewhere [18], A blood sample was collected in the morning after overnight fasting to measure fasting blood glucose (FBG), glycated hemoglobin (HbA1c), total cholesterol, high-density lipoprotein (HDL) cholesterol, and low-density lipoprotein (LDL) cholesterol and triglycerides.

### 2.3. Ophthalmological Assessment

**Figure 1:**
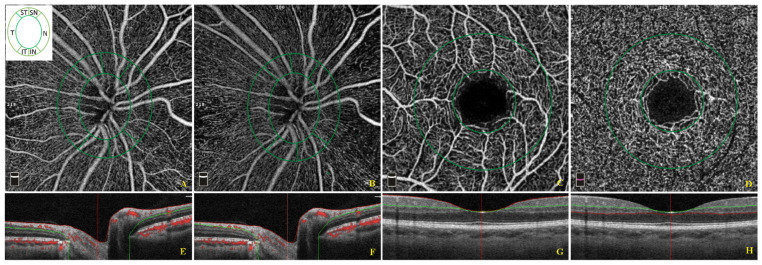
Optical coherence tomographic (OCT) angiographic images of the papillary region and macular region and corresponding structural OCT scans. The papillary vessel density measurement included measurements of the optic nerve head (ONH) (**A**) and radial peripapillary capillary (RPC) (**B**) plexi in an area of 4.5 × 4.5 mm^2^. The papillary area was subdivided into an optic disc area (inside optic disc, surrounded by the inner green circle) and six peripapillary regions (nasal, inferior nasal, inferior temporal, temporal, superior temporal, superior nasal) between the two green rings (**A**,**B**). The macular vessel density measurement included measurements of the superficial (**C**) and deep vascular (**D**) plexi in an area of 3 × 3 mm^2^. The macular area was divided into a foveal and parafoveal area between two concentric circles with a 1 mm diameter and 3 mm diameter, respectively (**C**,**D**). The colored lines (red and green) in horizontal OCT B-scans show segmentation lines defining the different depths in the retinal tissue. The ONH plexus is segmented from the inner limiting membrane to 150 μm below the inner limiting membrane (**E**). The RPC plexus is segmented from the upper boundary of the inner limiting membrane to the lower boundary of the nerve fiber layer (**F**). The superficial capillary plexus is segmented from approximately 3 μm below the inner limiting membrane to 15 μm below the inner plexiform layer (**G**). The deep capillary plexus is segmented from 15 μm below the inner plexiform layer to 70 μm below the inner plexiform layer (**H**).

Each participant underwent a complete ophthalmic examination including best-corrected visual acuity (BCVA) measurement, slit-lamp biomicroscopy, intraocular pressure (IOP) measurement, and funduscopy. BCVA was recorded as Snellen visual acuity and converted to the logarithm of minimal angle of resolution (LogMar) units for statistical analysis. Then, we performed OCT and OCT-A using Optovue RTVue XR 100 AVANTI, Optovue, Inc. (Fremont, CA, USA). OCT-A analyzes retinal vasculature after identification and segmentation of multiple retinal layers using the AngioVue module with Optovue RTVue AVANTI software (version 2015.100.0.35, Optovue, Inc., Fremont, CA, USA). The Angio Retina mode (3 × 3 mm^2^) and the Angio Disc (4.5 × 4.5 mm^2^) mode were employed. RTvue software includes the Optovue’s Motion Correction Technology (MCT™, Optovue, Inc., Fremont, CA, USA) and 3D Projection Artifact Technology. Furthermore, the software provided the signal strength index (SSI), which represents the scan’s reflectance signal strength, and a quality index (Q-score), representing the overall quality of the image, taking into account factors like SSI and motion artefacts [19]. In the present study, we only included images with a Q-score of 6 or above, SSI above 60, and without motion or shadow artefacts. The examinations were performed blinded by trained ophthalmologists. The vessel density (VD, %), defined as the percentage area occupied by the vessels in the corresponding region, was automatically measured by the built-in OCT device software. VD measurement was undertaken in the optic disc area, in the total peripapillary area, and in each of the six peripapillary sectors in two different layers, the ONH layer (Figure 1A,E) and the RPC layer (Figure 1B,F). Each layer corresponds with an en-face structural image. The software defines the peripapillary area as a 1.0 mm wide round annulus extending from the optic disc boundary and the inside optic disc area as a 2.0 mm diameter circle involving the optic disc. The peripapillary and the inside optic disc areas together composed the whole peripapillary area (4.0 mm-diameter round whole image). The peripapillary area, in turn, was divided into six peripapillary sectors. (Figure 1A,B,E,F). The software-provided peripapillary sectors are based on the Garway–Heath map [20]. The OCT angiograms centered on the fovea were automatically segmented to define the superficial plexus from 3 μm below the internal limiting membrane to 15 μm below the inner plexiform layer and the deep plexus from 15 to 70 μm below the inner plexiform layer. The VD at each macular plexus, superficial VD (SVD) and deep VD (DVD), was calculated for the whole 3 mm circle area centered on the fovea (whole retina), for the area between the outer 3 mm circle and the inner 1 mm circle (parafoveal quadrant), and for the area inside the central 1 mm circle (foveal quadrant) (Figure 1C,D,G,H).

The measurement of the foveal avascular zone (FAZ, mm^2^) at the deep capillary plexus (Figure S1) was performed as described in detail elsewhere [21]. The thickness (µm) of the GCC, composed of the thickness of RNFL, GCL and inner plexiform layer (IPL), at the macular area, and, separately, of the RNFL, were measured at the same time using the same OCT (Figure S2).

Exclusion criteria for all study participants were IOP > 22 mmHg, history of glaucoma, optic neuropathies, retinal diseases including any fundus findings suggesting hypertensive retinopathy (Grade 1–2 (Mild), 3 (Moderate), 4 (severe), according to the Keith–Wagener–Barker or Mitchell–Wong classification system) [22], a recent history of intraocular surgery, ocular trauma, and an obvious media opacity that could interfere with the OCT analysis.

### 2.4. Statistical Analyses

Continuous variables were expressed as mean ± standard deviation (SD) and categorical variables as proportion (%). Statistical significance was set with a *p*-value lesser than or equal to 0.05, with 95% confidence intervals (CI).

The whole sample was subdivided into two groups according to the presence or absence of hypertension. Due to the diffuse non-normal distribution of all variables (using the Shapiro distribution test), Wilcoxon rank sum test was performed to assess differences between the groups for continuous variables and Chi squared test for categorical variables. Logistic regression models were applied to assess associations between the unitary increases of OCT-A parameters that showed significant differences among the groups, as independent variables, and a hypertensive status as outcome. We built two hierarchical nested models: an unadjusted model and a fully adjusted model, adjusted for age, sex, BMI, MMSE, and IOP.

To reduce selection bias and simplify the reading of results we used a complete randomization algorithm for the eye selection assigning the corresponding value (left or right eye) to the new variable thus created. Moreover, we performed the Bonferroni corrected *p*-value in every model for every single OCT covariate.

Statistical analysis was performed with RStudio software, Version 1.4.1106 using additional packages: idyverse, randomizeR, rstatistix, Epi, kable.

## 3. Results

### 3.1. Descriptive Analysis

From 2016 to 2019, 892 of the 1929 participants in the Salus in Apulia Study underwent ophthalmological examinations. In addition, 124 subjects were excluded due to lack of data about hypertension, 20 due to glaucoma, 12 to hypertensive retinopathy, and 5 to erroneous scans including scans with segmentation failure. Overall, 731 participants were eligible for the final analysis presented in this study.

The average age of the participants was 73.4 ± 6.1 years, with a higher percentage of females (*n* = 434, 59.4%). The main sociodemographic and clinical characteristics of the whole sample, subdivided according to the presence/absence of hypertension, are shown in Table 1.

Older people with hypertension (higher SBP and DBP; *p* < 0.01, respectively) had a significantly greater waist circumference (*p* < 0.01) and BMI (*p* = 0.04). Moreover, the MCI prevalence was higher in subjects with hypertension (*p* < 0.01). Regarding blood tests, older people with hypertension had higher serum total cholesterol (*p* = 0.04), triglycerides levels (*p* = 0.02), Red Blood Cell count (*p* < 0.01), and hemoglobin (*p* < 0.01). Other sociodemographic characteristics of the whole population study, subdivided according to the presence/absence of hypertensive condition, not significantly different among the groups, are shown in the Table S1.

### 3.2. Analysis of Ophthalmological Parameters

Table 2 shows the ophthalmological parameters in older subjects with and without hypertension. BCVA and IOP were not significantly different between the groups.

The mean GCC thickness was slightly lower in patients with hypertension (*p* = 0.04). Mean RNFL thickness was slightly lower in the hypertension group than in subjects without hypertension with a significance rather close to the value 0.05 (*p* = 0.06). VD of ONH plexus at the inferior temporal sector was significantly lower in subjects with than without hypertension (*p* = 0.02). Conversely, VD of the ONH and RPC inside the optic disc was significantly higher in subjects with than without hypertension (*p* < 0.01). No significant difference was found between the groups regarding VD of ONH and RPC networks at the other sectors analyzed, FAZ area, and SVD and DVD at the foveal and parafoveal sites. However, all measurements of VD at the macular site revealed slightly higher values in hypertensive patients (Table S2).

### 3.3. Regression Models

The increase of GCC thickness was inversely associated with hypertension (OR: 0.98; 95% CI: 0.97–1). Also, the unitary increase of VD of the ONH plexus at the inferior temporal sector of the peripapillary area (OR:0.95, 95% CI: 0.91–0.99) was inversely associated with hypertensive condition. Conversely, the increase of VD of the ONH (OR:1.07, 95% CI: 1.04–1.10) and RPC inside Optic Disc (OR:1.04, 95% CI: 1.02–1.06) were directly associated with hypertension (Table 3).

All other OCT-A parameters not associated with hypertension in the models are reported in Table S3.

## 4. Discussion

In the present study conducted in an older population-based sample, on OCT, thinner GCC was observed in hypertensive subjects. On OCT-A, a capillary rarefaction at the inferior temporal sector of the ONH peripapillary plexus and, conversely, a higher density of the ONH and RPC network inside optic disc were significantly associated with hypertensive condition.

In our population-based sample, older people with hypertension had a higher BMI and waist circumference, suggesting the relationship between obesity and blood hypertension in older adults, as recently reported [23]. The MCI prevalence was also higher in hypertensive subjects, confirming the potential role of hypertension as risk factor for MCI (reduced function in memory, thinking, and other cognitive domains, but not affecting everyday functioning), as previously reported [24].

In the present study, the lowest average value for GCC and RNFL, was observed in older individuals with blood hypertension as compared to the control group. These findings were consistent with previous studies [6,10,11]. The reduced thickness of the inner neuroretinal layers observed, suggesting neural damage, was hypothesized to be related to microvascular abnormalities in patients with blood hypertension [10], similar to the proposed mechanism underlying the reduction of RNFL in diabetic patients without retinopathy [25]. An association with the hypertensive condition was mainly observed for GCC thickness, and this could be explained by the early damage of the GCL-IPL, containing ganglion cells bodies and dendrites, that precedes the damage of the axons in RNFL [26], as well as by the exclusion from the present study of patients with hypertensive retinopathy. On OCT-A, the larger difference in the average VD of the peripapillary networks between hypertensive patients and control subjects was reported at the inferior temporal sector of the ONH network and inside the optic disc of the RPC plexus. The vessels of ONH and RPC vascular networks have a relatively constant caliber and few anastomoses, paralleling the RNFL in the peripapillary area, which display a characteristic linear morphological pattern [27]. In healthy subjects, these networks are more prominent in the peripheral arcuate nerve fiber layer region, as well as in the temporal sectors, where the thinnest RNFL was located [27,28]. This opposite distribution could be due to the activity-vascular related mechanism by which denser temporal RPC exists to fulfill the highly metabolic requirements of photoreceptors, ganglion cells, and retinal pigment epithelial cells within the macular area [28], despite the reported association between RNFL thickness and peripapillary VD [29] supports the idea that the perfusion of RPC may be proportional to the quantity of RNFL supplied [12]. The peripapillary plexus is considered to be crucial for the homeostasis and function of the ganglion cells and their axons in the RNFL [30], and a reduced VD has been observed in early glaucoma [31] and non-arteritic ischemic optic neuropathy [32], associated with a reduced RNFL and GCC thickness [33]. Therefore, we may hypothesize that in the hypertensive condition the reduced thickness of GCC and RNFL could be related to the vascular rarefaction in a peripapillary sector with a high metabolic requirement. An increased vascular density inside the optic disc was recently observed in hypertensive subjects without related retinopathy [8,34], consistent with our finding. This is probably due to the increased reflux venous resistance related to hypertension vascular damage and the poor regulatory capacity of papillary ONH and RPC networks causing blood flow restriction and consequent vessels dilatation interpreted as an increased capillary density by OCT-A [8,34]. In contrast with previous results [8,11,34], we found a slightly lower vascular density of the macular superficial and deep vascular plexi and a slight enlargement of deep FAZ in normotensive individuals, with no significant difference as compared to hypertensive patients. Although these differences between the groups fall in the range of normal variation of macular vessel density measurements, as previously reported [35], they deserve some consideration. The mechanism underlying the association between blood hypertension and macular capillary density remains unclear. Previous studies have indicated a reduced retinal VD in hypertension as a possible effect of vascular narrowing due to an increased vascular resistance [4,11,36,37], which may impair blood flow. However, first of all, we should consider some technical limits of OCT-A whereby a slow blood flow above a minimum threshold detected by the machine may be wrongly reflected as a vascular rarefaction or even an area of non-perfusion on OCT-A images [37], and some optical parameters, such as the axial length of the eye, and in particular myopic condition, that could induce noise in the image, making the vascular network appear artificially denser because of the larger area being scanned under smaller magnification [38]. Furthermore, the results of 11 studies on macular vessels rarefaction reported in a recent meta-analysis [39] should be analyzed considering the Asiatic ethnicity of most of the study populations (9 out of 11 studies considered Asiatic populations) [8,10,11,34,40,41,42,43,44], and the inclusion of patients with hypertensive retinopathy in the study groups [10,44].

In the present study, the differences in VD between older subjects with and without hypertension showed a different trend at peripapillary and macular sites. Peripapillary vessels originate from two systems, the central retinal artery and the short posterior ciliary arteries, whereas macular vessels originate only from the central retinal artery [45]. The posterior ciliary arteries might suffer more severe damage than the retinal vascular system in glaucoma [46], diabetic retinopathy [15], and obstructive sleep apnea syndrome [45], because they are probably more prone to structural alterations due to high intraocular pressure, microangiopathy and hypercapnia, respectively. Therefore, the different origins and sizes of the vessels between the peripapillary and parafoveal areas might explain our findings, suggesting different damage to the vessels in different vascular systems attributable to hypertension.

The strengths of the present study included: a standardized measurement of daytime blood pressure in office setting, which might better reflect the hemodynamic load over the 24 h period than one single blood pressure measurement and could be more correlated with end-organ damage of arteries and heart [47,48]; OCT scanning combined with an ophthalmological clinical examination to avoid optical interferences due to ocular media abnormalities. Furthermore, we considered randomly the measurements of one eye for each subject as a good practice for statistical analysis [49] although, in all age groups, a moderate degree of interocular asymmetry in retinal layer thickness, including GCC and RNFL [50,51], and retinal vascular features [52], in both normotension and hypertension [53], was previously reported. However, some limitations and questions need to be considered. ONH parameters (rim, cup, etc.) were not analyzed in this study. We did not measure the ocular perfusion pressure as the net pressure gradient causing blood to flow to the eye, because of its limitations in reflecting the true perfusion pressure at the ONH, influenced by several variables as diurnal variations in the blood pressure and IOP [54], diurnal-to-nocturnal decreases in retinal and ONH blood flow in older patients [55], and intracranial pressure [56]. Moreover, the RPC plexus contains multilayered capillaries that are overlapping on en-face OCT images, complicating the ability to detect small capillary losses [31]. The macular deep vascular plexus is notoriously affected by projection artefacts which may lead to erroneous findings; one of these measures is the axial length, that was lacking in this study setting. Nor did we analyze in OCT-A the choriocapillaris, which appears as a mesh-like homogeneous tissue whose single vessels are usually not discernible. Measurement of the choriocapillaris network could be less precise than that of the intraretinal vascular networks [57]. A limit in methodology of the present study include the definition of the hypertensive status that was not based on previous clinical diagnosis as well as the presence of hypertensive medication, and the cross-sectional nature of the data, preventing assessment of the direction of the association, with a high risk of reverse causality bias.

In conclusion, the present findings confirmed a thinner GCC and microvascular changes at the peripapillary area in older hypertensive individuals than in age-matched healthy subjects. This conclusion and the higher MCI prevalence in hypertensive subjects suggests a link between central and peripheric neural damage with systemic vascular disregulation. Assessment of the peripapillary retinal microvasculature using OCT-A may be a useful non-invasive technique to detect early microvascular changes due to hypertension. Further larger studies, particularly with longitudinal cohort or randomized clinical trial designs, are needed to test the effectiveness of retinal capillary density as a novel biomarker in predicting the incidence and progression of hypertension-related microvascular complications, also at the population level.

## Figures and Tables

**Table 1:** Sociodemographic and clinical variables in subjects with and without hypertension. The Salus in Apulia Study (*n* = 731).

	Without Hypertension		With Hypertension		*p* *
	Mean ± SD/ Sample Size	Median (Min to Max)	Mean ± SD/ Sample Size	Median (Min to Max)	
**Sociodemographic Assessment**					
**Subjects (%)**	114 (15.6)		617 (84.4)		
**Age (years)**	73.2 ± 5.8	71.5 (65 to 89)	73.4 ± 6.1	72 (65 to 95)	0.93
**Sex**					0.23^χ2^
**Males (%)**	52 (45.6)	--	245 (39.7)	--	
**Females (%)**	62 (54.4)	--	372 (60.3)	--	
**Male/Female (%)**	83.9		65.9		
**Smokers**	9 (7.9)	--	36 (5.8)	--	0.40^χ2^
**Waist circumference (cm)**	99.7 ± 11.9	98 (70 to 127)	103.8 ± 10.2	104 (70 to 139)	**<0.01**
**BMI (kg/m^2^)**	27.3 ± 4.6	27.3 (18.4 to 43)	28.3 ± 4.7	27.9 (18.5 to 47.7)	**0.04**
**MMSE**	26.1 ± 3.9	27 (13 to 30)	26.5 ± 4.1	28 (1 to 30)	0.33
**MCI**	9 (7.9)	--	117 (19)	--	**<0.01**
**Metabolic Assessments**					
**SBP (mmHg)**	115.3 ± 6.4	120 (100 to 125)	136.1 ± 13	140 (100 to 180)	**<0.01**
**DBP (mmHg)**	68.2 ± 4.6	70 (50 to 75)	79.6 ± 6.7	80 (40 to 100)	**<0.01**
**HbA1c (mmol/mol)**	39.7 ± 9.5	39 (18 to 92)	39.6 ± 9.2	38 (19 to 128)	0.77
**HbA1c (%)**	5.8	5.7 (3.8 to 10.6)	5.8	5.6 (3.9 to 13.9)	
**Total cholesterol (mg/dL)**	178.4 ± 34.7	178 (85 to 270)	186.6 ± 37.2	186 (79 to 386)	**0.04**
**Triglycerides (mg/dL)**	93 ± 47.8	84.4 (17 to 292)	104.7 ± 55.6	92 (30 to 520)	**0.02**
**RBC (10^6^ cells/mm^3^)**	4.7 ± 0.5	4.6 (3.2 to 6.8)	4.8 ± 1.5	4.8 (2.9 to 40.8)	**<0.01**
**Hemoglobin (g/dL)**	13.4 ± 1.3	13.4 (9.5 to 16.9)	13.9 ± 1.5	13.9 (9 to 18.5)	**<0.01**

BMI: body mass index; MMSE: Mini Mental State Examination; MCI: mild cognitive impairment; SBP: systolic blood pressure; DBP diastolic blood pressure; HbA1c: glycated hemoglobin; RBC: Red Blood Cell. All data are shown as mean ± standard deviation (SD)/sample size, median (min to max) for continuous variables and as (%) for proportions. * Wilcoxon sum rank test; ^χ2^ Chi squared test.

**Table 2:** Ophthalmological variables in subjects with and without hypertension. The Salus in Apulia Study (*n* = 731).

	Without Hypertension		With Hypertension		
	Mean ± SD	Median (Min to Max)	Mean ± SD	Median (Min to Max)	*p* Value *
**BCVA (LogMar)**	0.13 ± 0.3	0.03 (0 to 1.6)	0.11 ± 0.2	0.03 (0 to 1.8)	0.70
**IOP (mmHg)**	14.9 ± 3.4	14.4 (10 to 22)	14.7 ± 3.1	14.5 (9 to 21)	0.14
**GCC thickness (µm)**	99.2 ± 18	96.5 (65 to 237.8)	95.4 ± 12.6	94.5 (44.7 to 180.1)	**0.04**
**RNFL thickness (µm)**	97.6 ± 10.7	98 (62 to 128)	95.5 ± 11	96 (57 to 127)	0.06
**ONH peripapillary inferior temporal VD (%)**	63.7 ± 4.8	64.5 (46.2 to 71.9)	62.2 ± 5.8	62.8 (38.9 to 72.3)	**0.02**
**ONH inside Optic Disc VD (%)**	58.6 ± 8.2	59.4 (28.7 to 72.6)	60.8 ± 6.4	62.1 (35.7 to 72.9)	**<0.01**
**RPC inside Optic Disc VD (%)**	38.7 ± 10.6	37.8 (13.3 to 67.2)	42.9 ± 10.8	43 (13.3 to 67.2)	**<0.01**
**SSI**	61.3 ± 10.4	62 (34 to 87.6)	62.6 ± 11.1	63.7 (2.1 to 87.6)	0.20

BCVA: best-corrected visual acuity; IOP: intraocular pressure; GCC: ganglion cell complex; RNFL: retinal nerve fiber layer; ONH: optic nerve head; VD: vascular density; RPC: radial peripapillary capillary; SSI, signal strength index; * Wilcoxon sum rank test.

**Table 3:** Logistic regression models on hypertension status (Yes/No) as dependent variable and regressors. N: 731.

	Raw Model			Adjusted Model				
	OR	CI 95%	Stand. Err.	OR	CI 95%	Stand. Err.	*p*	Adj. *p*
**GCC Thickness (µm)**	**0.98**	**0.97 to 0.99**	**0.01**	**0.98**	**0.97 to 0.99**	**0.01**	**0.01**	**0.04**
Age (years)				1	0.97 to 1.04	0.01	0.58	0.99
Sex (Female)				1.26	0.83 to 1.82	0.21	0.42	0.99
BMI (Kg/m^2^)				1.04	1.00 to 1.09	0.02	0.06	0.36
IOP				1.07	0.96 to 1.19	0.05	0.21	0.99
MMSE				1.03	0.98 to 1.09	0.02	0.22	0.99
**ONH peripapillary** **Inferior Temporal VD (%)**	**0.95**	**0.91 to 0.99**	**0.02**	**0.95**	**0.91 to 0.99**	**0.02**	**0.01**	**0.05**
Age (years)				1.01	0.97 to 1.05	0.01	0.61	0.99
Sex (Female)				1.17	0.77 to 1.79	0.21	0.45	0.99
BMI (Kg/m^2^)				1.04	1.00 to 1.09	0.02	0.07	0.49
IOP				1.07	0.96 to 1.19	0.05	0.20	0.99
MMSE				1.04	0.98 to 1.09	0.02	0.18	0.99
**ONH Inside Optic Disc VD (%)**	**1.06**	**1.03 to 1.10**	**0.01**	**1.07**	**1.04 to 1.10**	**0.06**	**<0.01**	**<0.01**
Age (years)				1.02	0.98 to 1.06	0.02	0.27	0.99
Sex (Female)				1.22	0.80 to 1.88	0.20	0.34	0.99
BMI (Kg/m^2^)				1.04	0.99 to 1.09	0.04	0.06	0.42
Mean IOP				1.07	0.96 to 1.19	0.07	0.18	0.99
MMSE				1.02	0.97 to 1.08	0.02	0.32	0.99
**RPC Inside Optic Disc VD (%)**	**1.04**	**1.02 to 1.05**	**0.01**	**1.04**	**1.02 to 1.06**	**0.01**	**<0.01**	**<0.01**
Age (years)				1.03	0.99 to 1.07	0.01	0.18	0.99
Sex (Female)				1.11	0.72 to 1.71	0.21	0.63	0.99
BMI (Kg/m^2^)				1.04	0.99 to 1.09	0.02	0.08	0.56
IOP				1.09	0.98 to 1.21	0.05	0.10	0.70
MMSE				1.04	0.98 to 1.09	0.02	0.17	0.99

GCC: ganglion cell complex; BMI: Body Mass Index; IOP: intraocular pressure; MMSE: Mini Mental State Evaluation; ONH: optic nerve head; VD: vascular density; RPC: radial peripapillary capillary.

## Data Availability

The data that support the findings of the present study are available from the corresponding author (GS) upon reasonable request.

## Supplementary Materials

The following supporting information can be downloaded at: https://www.mdpi.com/article/10.3390/jcm11041098/s1, Table S1. Sociodemographic and clinical variables in subjects with and without hypertension. The Salus in Apulia Study (*n* = 731). Table S2. Ophthalmological variables in subjects with and without hypertension. The Salus in Apulia Study (*n* = 731). Table S3. Logistic regression models on Hypertension status (Yes/No) as dependent variable and regressors. N:731. Figure S1. Optical coherence tomographic angiographic (OCT-A) image of the foveal avascular zone (FAZ) areas at the deep vascular plexus. The FAZ area border (inner yellow line) was outlined automatically, and the surface area was then measured in square millimeters using built-in software under the flow option of the instrument in the deep vascular plexus (A) The colored lines (red and green) in horizontal OCT B-scans show segmentation lines defining the depths in the retinal tissue (B). Figure S2. Optical coherence tomographic images of the ganglion cell complex (GCC) and retinal nerve fiber layer (RNFL) thickness. The device measures GCC and RNFL thickness within an automatically rendered 7 mm^2^ area, centered 1 mm temporally to the fovea. The system produces a color-coded thickness map for interpretation. (A,C) Thicker regions of GCC and RNFL are displayed as yellow and orange, whereas thinner regions are displayed as blue and green. Acquired thicknesses are compared with values from a normative database and displayed as a significance map. The color-coded map shows the corresponding probabilities of deviation from the normal range based on comparison with an age-matched control group of healthy subjects. (B,D) Cross-sectional B-scan displays the segmentation used for GCC and RNFL analysis. (B) The traced boundaries for the GCC scan (white lines) include the inner limiting membrane and the outer IPL. (D) The traced boundaries for the RNFL scan (colored lines) include the inner limiting membrane and the outer IPL.
