# Supplementary material for: Association of Neuroretinal Thinning and Microvascular Changes with Hypertension in an Older Population in Southern Italy
Source: J Clin Med. 2022 Feb 19;11(4):1098. doi: 10.3390/jcm11041098 (PMC8879471; doi:10.3390/jcm11041098)
Supplement: Supplementary file 1 [file jcm-11-01098-s001.zip › jcm-1569421-supplementary.pdf]

Table S1. Sociodemographic and clinical variables in subjects with and without hypertension. The Salus in Apulia Study (*n* = 731).

	Without Hypertension		With Hypertension		
	**mean ± sd**	**median (min to max)**	**mean ± sd**	**median (min to max)**	**p value ***
**Education (years)**	6.85 ±3.72	5 (0 to 18)	7.25 ±3.85	5 (0 to 18)	0.19
**Apathy Evaluation Scale**	5.53 ±5.82	4 (0 to 26)	4.52 ±4.91	3 (0 to 30)	0.09
**Late-life depression**	19 (18.30)	--	66 (12.00)	--	0.06
**Physical frailty**	24 (21.10)	--	141 (22.90)	--	$0.66^{\chi 2}$
**Frontal Assessment Battery**	12.87 ±3.76	13 (4 to 18)	13.15 ±3.67	14 (0 to 18)	0.44
**FBG (mg/dl)**	102.2 ±23.12	96.5 (79 to 239)	104.2 ±22.96	99 (59 to 300)	0.06
**WBC (10^3 cells/mm^3)**	5.97 ±1.71	5.67 (1.55 to 11.09)	6.2 ±2.05	5.93 (2.57 to 30.88)	0.31
**Platelets (10^3 cells/mm^3)**	223.1 ±50.3	215.5 (64 to 379)	226.76 ±58.81	221 (65 to 563)	0.87
**HDL cholesterol (mg/dl)**	48.36±12.5	45.5 (26 to 89)	49.43±15.56	47 (22 to 265)	0.49
**LDL cholesterol (mg/dl**	110.97±29.6	110 (41 to 198	115.74±31.73	115 (28 to 220)	0.15
**IL 6 (pg/ml)**	3.43 ±5.29	1.89 (0.24 to 39.3)	3.41 ±6.25	1.87 (0.06 to 64.94)	0.86
**CRP (mg/dl)**	0.64 ±1.12	0.36 (0.1 to 10.96)	0.52 ±0.65	0.31 (0.1 to 9.72)	0.38

FBG: fasting blood glucose; WBC: white blood cell; HDL: high-density lipoprotein; LDL: low-density lipoprotein. All data are show as mean ± sd, median (min to max) for continuous variables and as n (%) for proportions. * Wilcoxon sum rank test; $\chi 2$ Chi squared test.

Table S2. Ophthalmological variables in subjects with and without hypertension. The Salus in Apulia Study (n = 731).

	Without Hypertension		With Hypertension		
	**Mean ± sd**	**Median (Min to Max)**	**Mean ± sd**	**Median (Min to Max)**	***p* Value ***
**ONH Whole peripapillary VD (%)**	53.62 ± 3.69	54.30 (42.17 to 59.89)	53.76 ± 3.83	54.30 (27.73 to 62.72)	0.73
**ONH Peripapillary area VD (%)**	59.54 ± 2.88	59.69 (51.55 to 64.99)	59.09 ± 3.21	59.30 (36.65 to 67.36)	0.11
**ONH Nasal VD (%)**	58.30 ± 5.40	59.92 (40.23 to 66.2)	58.08 ± 5.28	59.16 (33.73 to 66.73)	0.46
**ONH Superior Nasal VD (%)**	60.84 ± 7.81	61.45 (39.82 to 73.95)	61.25 ± 7.06	62.08 (37.54 to 73.95)	0.66
**ONH Inferior Nasal VD (%)**	60.81 ± 6.73	61.12 (43.2 to 74.5)	49.42 ± 6.23	49.89 (36.2 to 66.2)	0.74
**ONH Temporal VD (%)**	58.71 ± 6.21	59.13 (38.2 to 68.77)	58.65 ± 6.12	59.29 (38.2 to 68.77)	0.87
**ONH Superior Temporal VD (%)**	57.33 ± 8.51	57.91 (26.5 to 69.61)	56.66 ± 9.09	58.06 (16.76 to 69.61)	0.67
**RPC Whole peripapillary VD (%)**	52.49 ± 3.55	53.13 (42.33 to 59.99)	52.54 ± 4.11	52.98 (23.79 to 60.7)	0.85

**RPC Peripapillary area VD (%)**	60.42 ± 3.61	60.85 (44.71 to 68.59)	60.02 ± 3.85	60.41 (33.65 to 72.27)	0.33
**RPC Nasal VD (%)**	56.18 ± 5.82	56.45 (39.17 to 67.7)	56.19 ± 5.69	56.47 (24.08 to 69.38)	0.80
**RPC Inferior Nasal VD (%)**	61.42 ± 8.02	63.64 (43.06 to 73.22)	61.54 ± 8.12	63 (35.15 to 75.78)	0.90
**RPC Superior Nasal VD (%)**	64.81 ± 6.95	65.81 (38.57 to 77.23)	63.93 ± 7.13	65.36 (20.03 to 77.23)	0.22
**RPC Temporal VD (%)**	62.02 ± 5.29	61.83 (44.89 to 72.23)	61.29 ± 6.15	61.55 (24.32 to 72.56)	0.36
**RPC Superior Temporal VD (%)**	51.68 ± 5.87	52.77 (36.3 to 64.4)	57.56 ± 8.50	59.48 (28.7 to 72.6)	0.28
**RPC Inferior Temporal VD (%)**	65.53 ± 6.22	66.14 (44.53 to 78.14)	64.45 ± 6.96	65.66 (37.54 to 78.14)	0.18
**FAZ (mm^2)**	0.32 ± 0.27	0.31 (0.04 to 2.65)	0.29 ±0.23	0.28 (0.02 to 4.7)	0.13
**DVD Foveal (%)**	27.09 ± 7.04	26.09 (10.93 to 59.1)	27.68 ± 7.49	26.93 (6.27 to 74.88)	0.43

**DVD ParaFoveal (%)**	57.32 ± 6.61	58.78 (22.74 to 66.13)	57.42 ± 5.12	58.58 (28.18 to 66.71)	0.75
**DVD Whole(%)**	54.23 ± 6.07	55.17 (21.32 to 62.5)	54.57 ± 4.85	55.74 (29.51 to 63.29)	0.81
**SVD Foveal (%)**	29.47 ± 7.46	28.23 (10.62 to 59.15)	29.52 ± 6.35	29.22 (12.75 to 61.31)	0.68
**SVD ParaFoveal (%)**	51.77 ± 5.27	53.23 (32.84 to 60.14)	52.05 ± 4.78	53.11 (30.25 to 60.71)	0.85
**SVD Whole (%)**	49.76 ± 4.08	50.45 (36.77 to 57.62)	50.16 ± 4.16	50.87 (33.05 to 58.51)	0.24

ONH: optic nerve head; VD: vascular density; RPC: radial peripapillary capillary; DVD: deep vascular density; SVD: superficial vascular density; * *Wilcoxon sum rank test.*

Table S3. Logistic regression models on Hypertension status (Yes/No) as dependent variable and regressors. N:731.

	Raw Model			Adjusted Model		
	OR	CI 95%	Stand. Err.	OR	CI 95%	Stand. Err.
SSI	1.01	0.99 to 1.03	0.02	1.01	0.99 to 1.03	0.01
Age (years)				1.02	0.99 to 1.06	0.01
Sex (Female)				1.18	0.77 to 1.80	0.16
BMI (Kg/m²)				1.04	1.00 to 1.09	0.04
Mean IOP				1.07	0.97 to 1.19	0.05
MMSE				1.03	0.98 to 1.09	0.02
FAZ (mm²)	0.66	0.34 to 1.29	0.34	0.64	0.33 to 1.27	0.34
Age (years)				1.01	0.98 to 1.05	0.01
Sex (Female)				1.20	0.78 to 1.83	0.21
BMI (Kg/m²)				1.04	1.00 to 1.09	0.02
Mean IOP				1.07	0.97 to 1.19	0.05
MMSE				1.03	0.98 to 1.09	0.02
ONH Nasal VD (%)	0.99	0.95 to 1.03	0.03	0.99	0.95 to 1.03	0.03
Age (years)				1.01	0.97 to 1.05	0.01
Sex (Female)				1.20	0.79 to 1.84	0.21
BMI (Kg/m²)				1.04	1.00 to 1.09	0.02
Mean IOP				1.07	0.97 to 1.19	0.05
MMSE				1.04	0.98 to 1.09	0.02
ONH Peripapillary VD (%)	0.95	0.89 to 1.02	0.01	0.95	0.89 to 1.02	0.03
Age (years)				1.01	0.97 to 1.05	0.01
Sex (Female)				1.21	0.79 to 1.85	0.21
BMI (Kg/m²)				1.04	0.99 to 1.09	0.02
Mean IOP				1.07	0.97 to 1.19	0.05
MMSE				1.04	0.98 to 1.09	0.02
ONH Superior Nasal VD (%)	1.01	0.98 to 1.04	0.01	1.00	0.98 to 1.03	0.01
Age (years)				1.01	0.97 to 1.05	0.01
Sex (Female)				1.20	0.79 to 1.83	0.21
BMI (Kg/m²)				1.04	1.00 to 1.09	0.02
Mean IOP				1.07	0.97 to 1.19	0.05
MMSE				1.03	0.98 to 1.09	0.02
ONH Superior TemporalVD(%)	0.99	0.97 to 1.01	0.01	1.00	0.97 to 1.03	0.01
Age (years)				1.01	0.97 to 1.05	0.01
Sex (Female)				1.20	0.79 to 1.83	0.21
BMI (Kg/m²)				1.04	1.00 to 1.09	0.02
Mean IOP				1.07	0.97 to 1.19	0.05
MMSE				1.03	0.98 to 1.09	0.02
ONH Temporal VD (%)	1.00	0.97 to 1.03	0.02	1.00	0.97 to 1.03	0.01
Age (years)				1.01	0.97 to 1.05	0.01
Sex (Female)				1.20	0.79 to 1.83	0.21
BMI (Kg/m²)				1.04	1.00 to 1.09	0.02
Mean IOP				1.07	0.97 to 1.19	0.05
MMSE				1.03	0.98 to 1.09	0.02
ONH Whole VD (%)	1.01	0.96 to 1.06	0.02	1.02	0.97 to 1.07	0.02
Age (years)				1.01	0.98 to 1.05	0.01
Sex (Female)				1.21	0.79 to 1.85	0.21
BMI (Kg/m²)				1.04	1.00 to 1.09	0.02
Mean IOP				1.07	0.97 to 1.19	0.05
MMSE				1.03	0.98 to 1.09	0.02
RPC Inferior Nasal VD(%)	1.00	0.98 to 1.03	0.01	1.00	0.97 to 1.00	0.01
Age (years)				1.01	0.97 to 1.05	0.01
Sex (Female)				1.20	0.79 to 1.84	0.21
BMI (Kg/m²)				1.04	1.00 to 1.09	0.02
Mean IOP				1.07	0.97 to 1.19	0.05
MMSE				1.03	0.98 to 1.09	0.02
RPC Inferior Temporal VD (%)	0.98	0.95 to 1.01	0.01	0.98	0.95 to 1.01	0.01
Age (years)				1.01	0.97 to 1.05	0.01
Sex (Female)				1.21	0.79 to 1.85	0.21
BMI (Kg/m²)				1.04	1.00 to 1.09	0.02
Mean IOP				1.07	0.96 to 1.19	0.05
MMSE				1.03	0.98 to 1.09	0.02
RPC Nasal VD (%)	1.01	0.97 to 1.04	0.01	1.01	0.97 to 1.04	0.01
Age (years)				1.01	0.97 to 1.05	0.01
Sex (Female)				1.20	0.79 to 1.84	0.21
BMI (Kg/m²)				1.04	1.00 to 1.09	0.02
Mean IOP				1.07	0.97 to 1.19	0.05
MMSE				1.03	0.98 to 1.09	0.02
RPC Peripapillary VD (%)	0.97	0.92 to 1.03	0.02	0.97	0.92 to 1.03	0.02
Age (years)				1.01	0.97 to 1.05	0.01
Sex (Female)				1.20	0.79 to 1.84	0.21
BMI (Kg/m²)				1.04	1.00 to 1.09	0.02
Mean IOP				1.07	0.97 to 1.19	0.05
MMSE				1.04	0.98 to 1.09	0.02
RPC Superior Temporal VD (%)	0.98	0.96 to 1.01	0.23	0.98	0.96 to 1.01	0.01
Age (years)				1.01	0.97 to 1.05	0.01
Sex (Female)				1.19	0.78 to 1.83	0.18
BMI (Kg/m²)				1.04	0.99 to 1.09	0.04
Mean IOP				1.07	0.96 to 1.19	0.07
MMSE				1.03	0.98 to 1.09	0.03

	Raw Model			Adjusted Model		
	OR	CI 95%	Stand. Err	OR	CI 95%	Stand. Err
RPC Superior Nasal VD %	0.98	0.95 to 1.01	0.01	0.98	0.95 to 1.01	0.01
Age (years)				1.01	0.98 to 1.05	0.01
Sex (Female)				1.21	0.79 1.85	0.21
BMI (Kg/m²)				1.04	0.99 to 1.09	0.02
Mean IOP				1.07	0.97 to 1.19	0.05
MMSE				1.04	0.98 to 1.09	0.02
RPC Temporal VD (%)	0.98	0.95 to 1.01	0.01	0.98	0.95 to 1.01	0.01
Age (years)				1.01	0.98 to 1.05	0.01
Sex (Female)				1.21	0.79 to 1.85	0.21
BMI (Kg/m²)				1.04	0.99 to 1.09	0.02
Mean IOP				1.07	0.97 to 1.19	0.05
MMSE				1.04	0.98 to 1.09	0.02
RPC Whole VD (%)	1.00	0.95 to 1.06	0.02	1.01	0.96 to 1.07	0.02
Age (years)				1.01	0.98 to 1.05	0.01
Sex (Female)				1.20	0.79 to 1.83	0.21
BMI (Kg/m²)				1.04	1.00 to 1.09	0.02
Mean IOP				1.08	0.97 to 1.19	0.05
MMSE				1.03	0.98 to 1.09	0.02
RNFL Thickness	0.98	0.96 to 1.10	0.01	0.98	0.97 to 1.00	0.01
Age (years)				1.01	0.97 to 1.05	0.01
Sex (Female)				1.20	0.78 to 1.83	0.21
BMI (Kg/m²)				1.04	1.00 to 1.09	0.02
Mean IOP				1.07	0.96 to 1.19	0.05
MMSE				1.03	0.98 to 1.09	0.02
SVD Whole	1.02	0.98 to 1.07	0.02	1.03	0.98 to 1.09	0.02
Age (years)				1.02	0.98 to 1.06	0.01
Sex (Female)				1.19	0.78 to 1.82	0.21
BMI (Kg/m²)				1.04	1.00 to 1.09	0.02
Mean IOP				1.08	0.97 to 1.20	0.05
MMSE				1.03	0.98 to 1.09	0.02
SVD Foveal	1	0.96 to 1.04	0.02	1.00	0.96 to 1.05	0.02
Age (years)				0.99	0.95 to 1.05	0.02
Sex (Female)				1.34	0.75 to 2.37	0.29
BMI (Kg/m²)				1.05	0.99 to 1.12	0.03
Mean IOP				1.00	0.87 to 1.14	0.06
MMSE				1.07	1.01 to 1.15	0.03
SVD ParaFoveal	1.01	0.97 to 1.05	0.02	1.02	0.98 to 1.06	0.01
Age (years)				1.02	0.98 to 1.05	0.01
Sex (Female)				1.21	0.79 to 1.84	0.18
BMI (Kg/m²)				1.04	1.00 to 1.09	0.04
Mean IOP				1.07	0.97 to 1.19	0.07
MMSE				1.03	0.98 to 1.09	0.03
DVD Whole	1.01	0.98 to 1.05	0.01	1.02	0.98 to 1.06	0.02
Age (years)				1.02	0.98 to 1.06	0.01
Sex (Female)				1.20	0.78 to 1.83	0.17
BMI (Kg/m²)				1.04	1.00 to 1.09	0.04
Mean IOP				1.08	0.97 to 1.19	0.07
MMSE				1.03	0.98 to 1.09	0.03
DVD Foveal	1.01	0.98 to 1.04	0.01	1.01	0.98 to 1.04	0.01
Age (years)				1.01	0.97 to 1.05	0.01
Sex (Female)				1.19	0.78 to 1.82	0.21
BMI (Kg/m²)				1.04	1.00 to 1.09	0.02
Mean IOP				1.07	0.97 to 1.19	0.05
MMSE				1.04	0.98 to 1.09	0.02
DVD ParaFoveal	1.01	0.97 to 1.04	0.01	1.02	0.98 to 1.06	0.01
Age (years)				1.02	0.98 to 1.06	0.01
Sex (Female)				1.20	0.79 to 1.83	0.21
BMI (Kg/m²)				1.04	1.00 to 1.09	0.02
Mean IOP				1.07	0.97 to 1.19	0.05
MMSE				1.03	0.98 to 1.09	0.02
ONH Inferior Nasal VD (%)	1.01	0.97 to 1.03	0.01	0.99	0.96 to 1.03	0.01
Age (years)				1.101	0.97 to 1.05	0.01
Sex (Female)				1.20	0.78 to 1.84	0.21
BMI (Kg/m²)				1.104	0.99 to 1.09	0.02
Mean IOP				1.07	0.96 to 1.19	0.05
MMSE				1.03	0.98 to 1.09	0.02

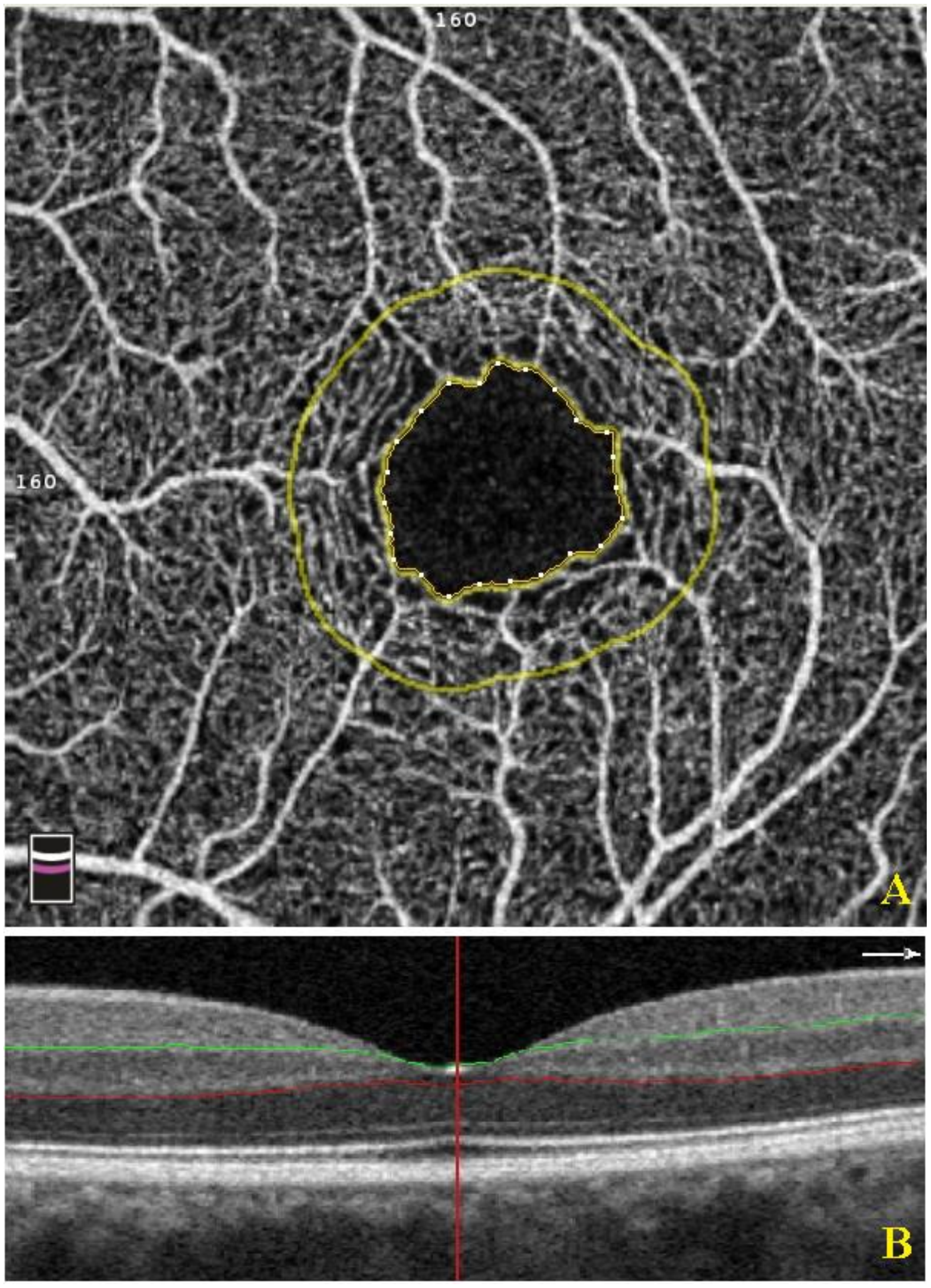

Figure S1. Optical coherence tomographic angiographic (OCT-A) image of the foveal avascular zone (FAZ) areas at the deep vascular plexus. The FAZ area border (inner yellow line) was outlined automatically, and the surface area was then measured in square millimeters using built-in software under the flow option of the instrument in the deep vascular plexus (**A**) The colored lines (red and green) in horizontal OCT B-scans show segmentation lines defining the depths in the retinal tissue (**B**).

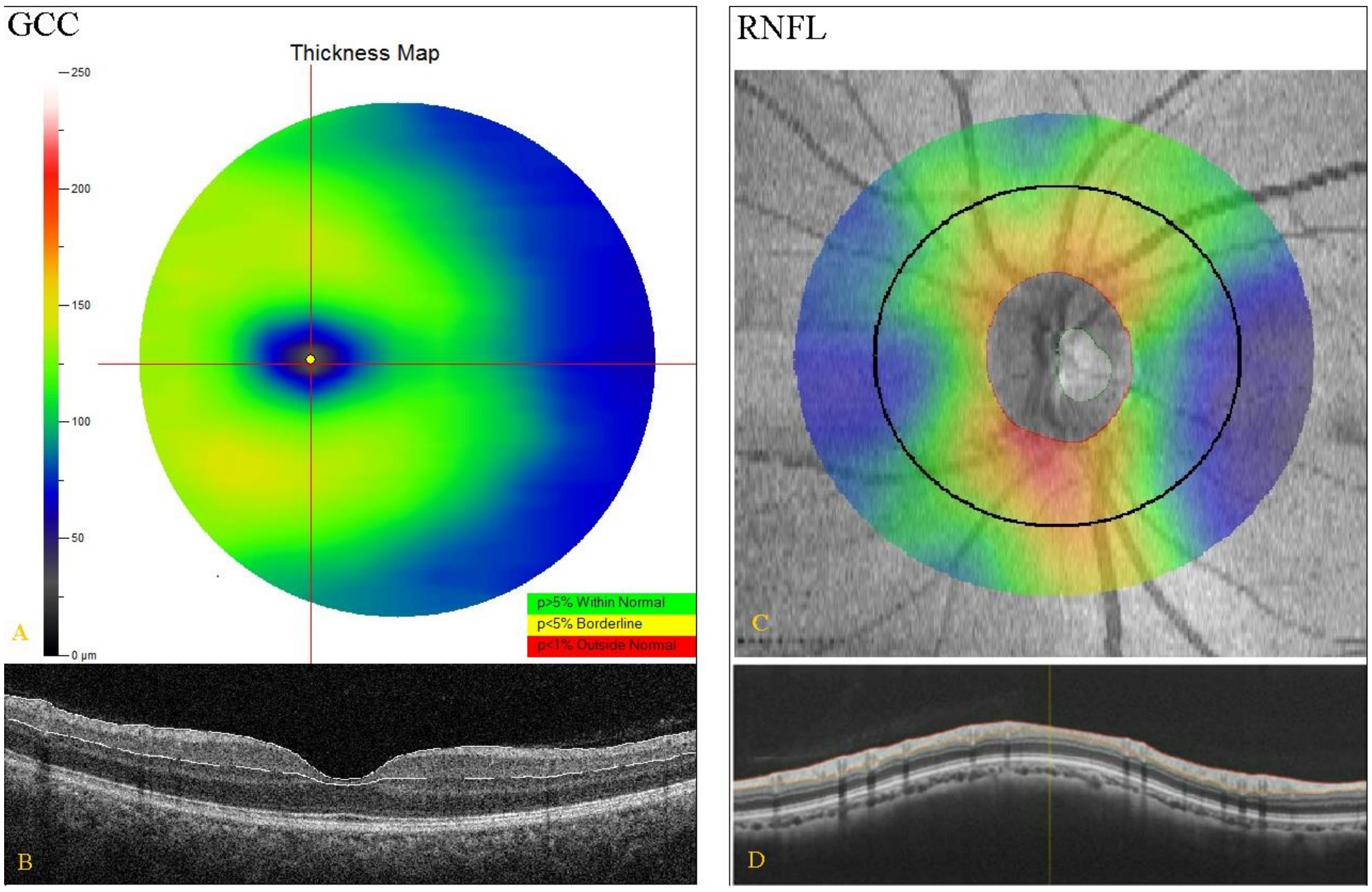

Figure S2. Optical coherence tomographic images of the ganglion cell complex (GCC) and retinal nerve fiber layer (RNFL) thickness. The device measures GCC and RNFL thickness within an automatically rendered 7-mm2 area, centered 1 mm temporally to the fovea. The system produces a color-coded thickness map for interpretation. (**A**,**C**) Thicker regions of GCC and RNFL are displayed as yellow and orange, whereas thinner regions are displayed as blue and green. Acquired thicknesses are compared with values from a normative database and displayed as a significance map. The color-coded map shows the corresponding probabilities of deviation from the normal range based on comparison with an age-matched control group of healthy subjects. (**B**,**D**) Cross-sectional B-scan displays the segmentation used for GCC and RNFL analysis. (**B**) The traced boundaries for the GCC scan (white lines) include the inner limiting membrane and the outer IPL. (**D**) The traced boundaries for the RNFL scan (colored lines) include the inner limiting membrane and the outer IPL.
